# Supplementary material for: Dealumination and Characterization of Natural Mordenite-Rich Tuffs
Source: Materials (Basel). 2022 Jul 1;15(13):4654. doi: 10.3390/ma15134654 (PMC9267643; doi:10.3390/ma15134654)
Supplement: Supplementary file 1 [file materials-15-04654-s001.zip › materials-1724340-supplementary.pdf]

Dealumination of Natural Mordenite and Its Potential Use as Catalyst Support

Armando Adriano[1,2], Mauricio Cornejo[1,2*], Haci Baykara[1,2], Eduardo V. Ludeña[1,3], Joaquín L. Brito[3]

[1] Escuela Superior Politécnica del Litoral, ESPOL, Center of Research and Development in Nanotechnology, CIDNA, Km 30.5 vía Perimetral, Campus G. Galindo, Guayaquil, Ecuador.

[2] Escuela Superior Politécnica del Litoral, ESPOL, Facultad de Ingeniería Mecánica y Ciencias de la Producción, FIMCP, Km 30.5 via Perimetral, Campus Gustavo Galindo, Guayaquil, Ecuador.

[3] Laboratorio de Fisicoquíımica de Superficies, Centro de Quíımica, Instituto Venezolano de Investigaciones Científicas, I.V.I.C. Apartado 2182.

[*] Corresponding author: M.Cornejo, mcornejo@espol.edu.ec

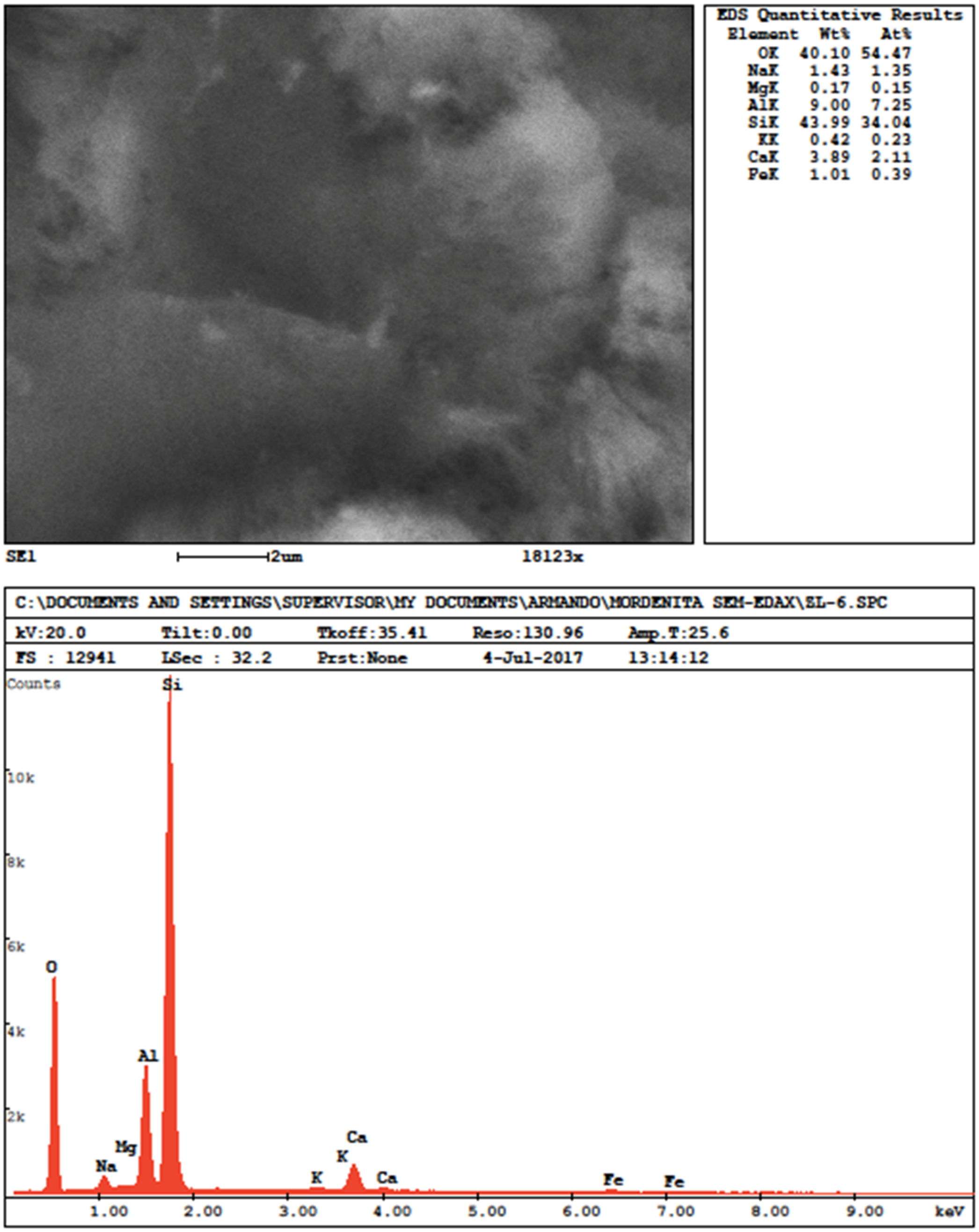

Figure S1. SEM-EDS analysis of ZL sample.

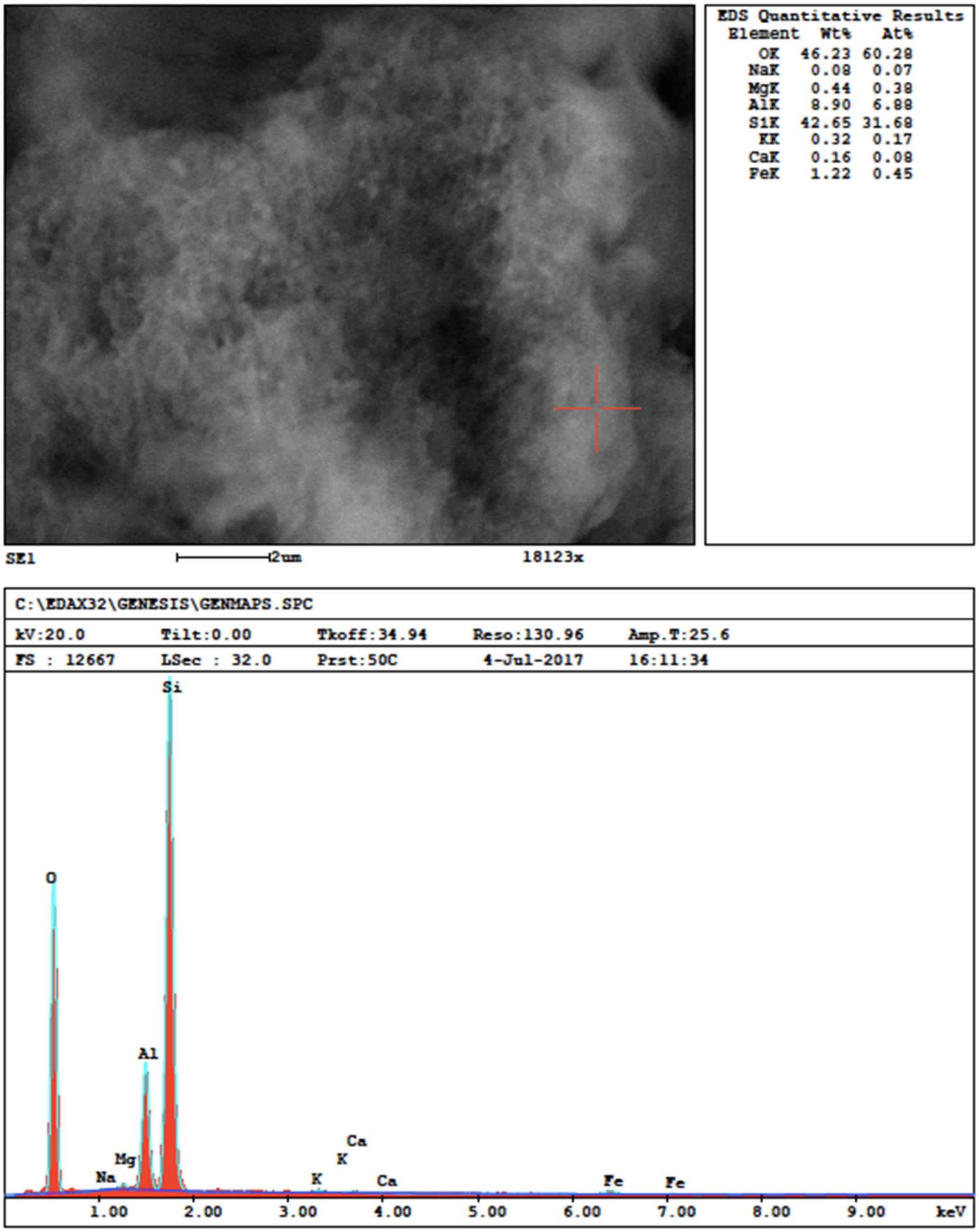

Figure S2. SEM-EDS analysis of ZAM sample.

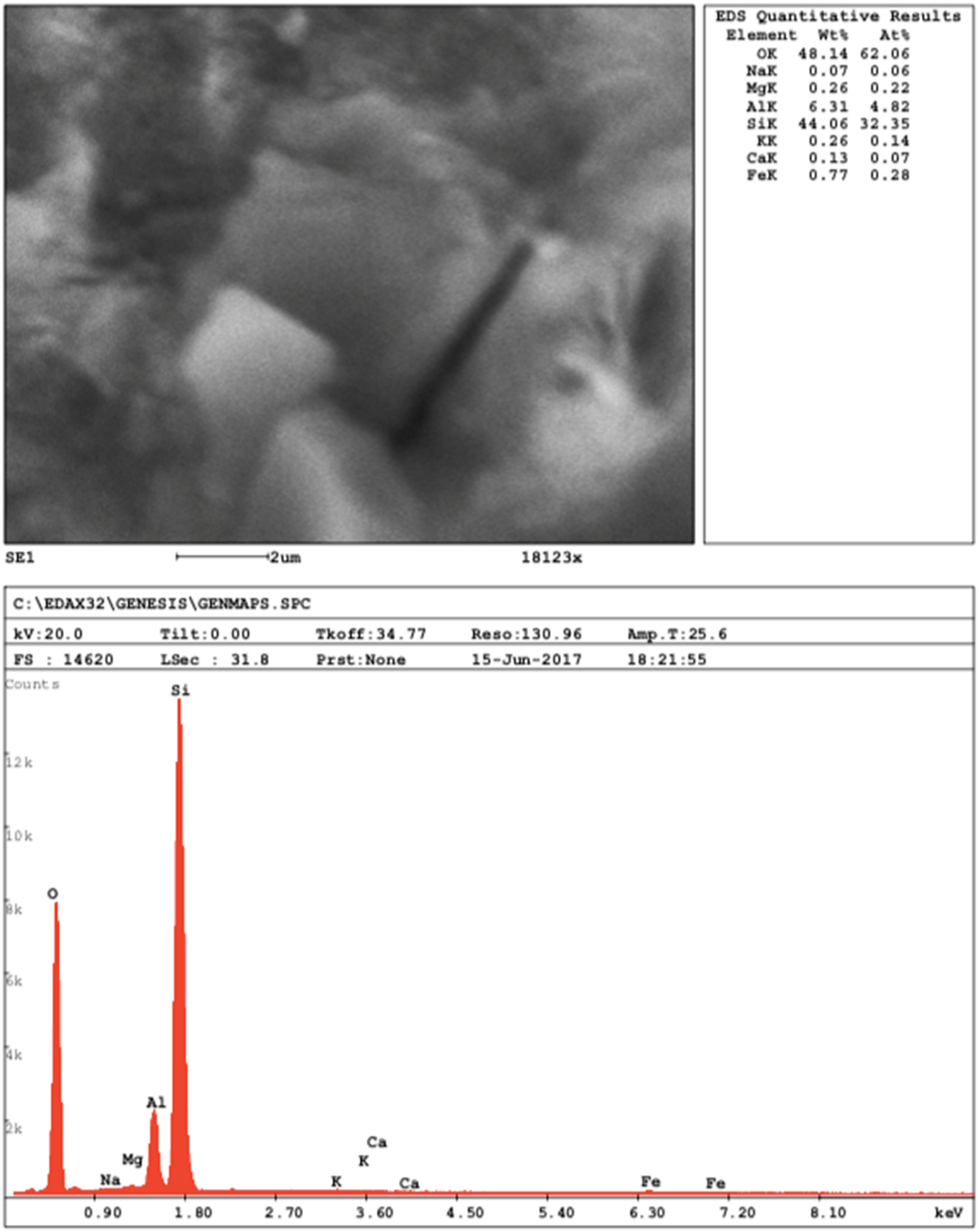

Figure S3. SEM-EDS analysis of ZDES1 sample.

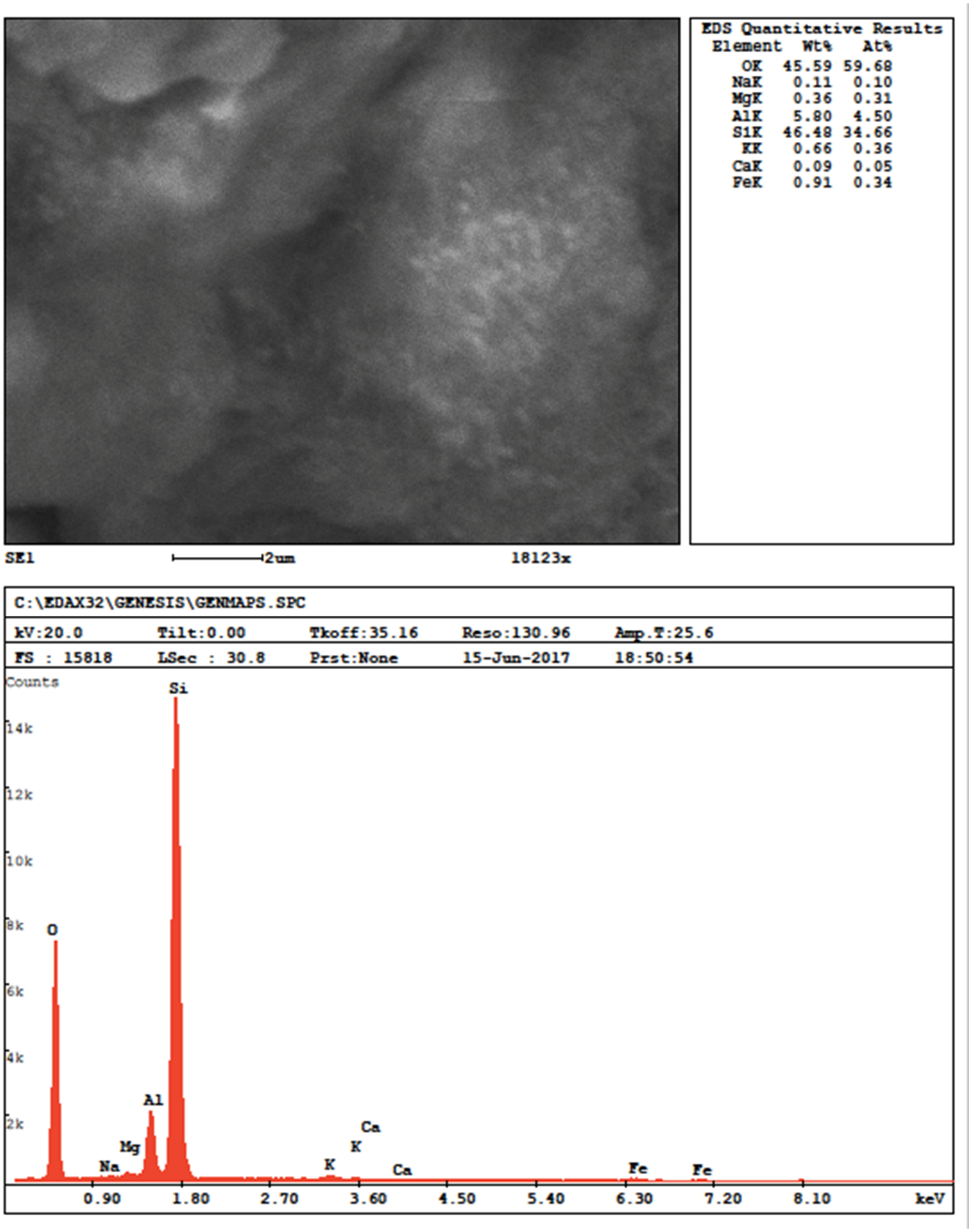

Figure S4. SEM-EDS analysis of ZDES3 sample.

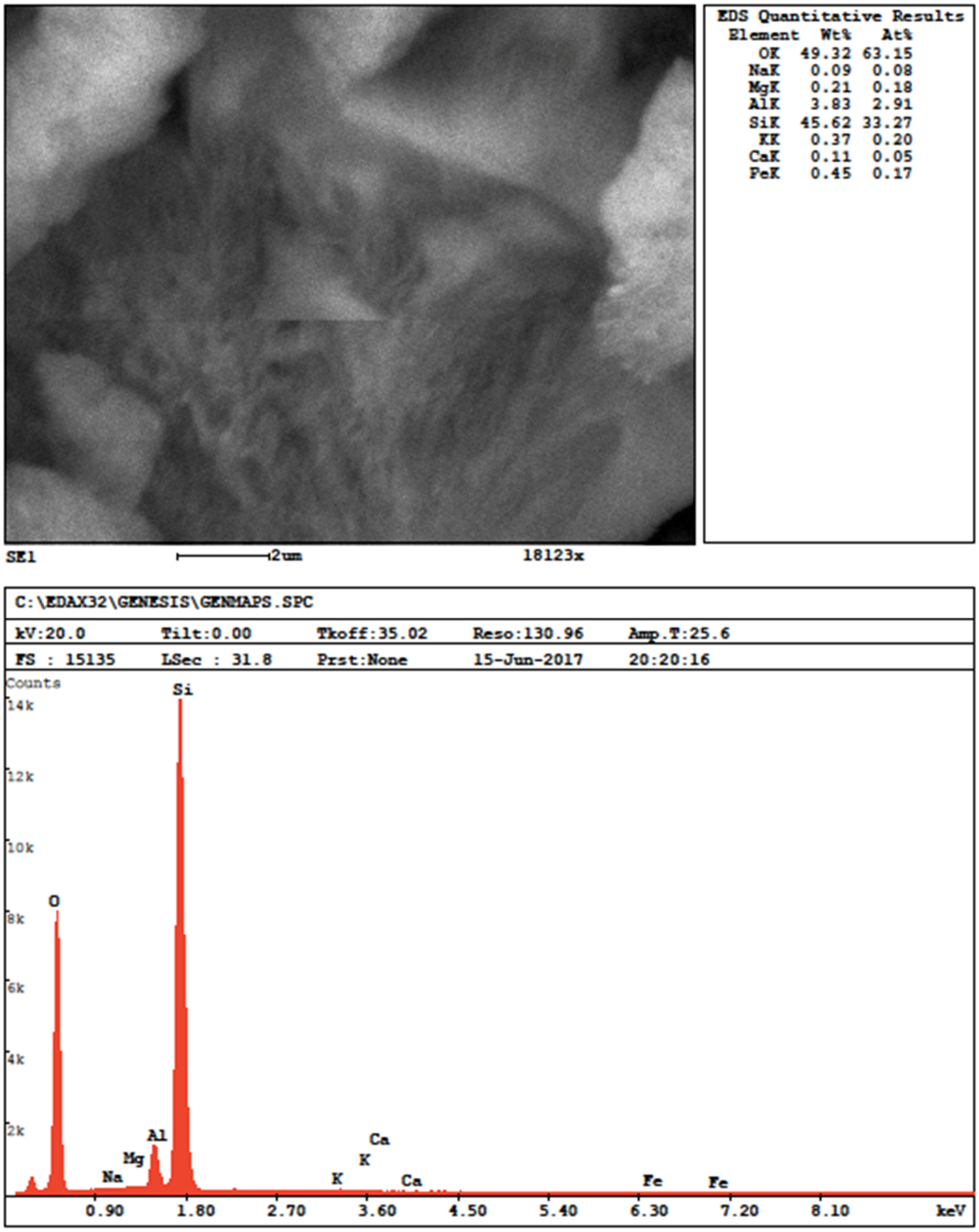

Figure S5. SEM-EDS analysis of ZDES5 sample.
